# The evolutionary origins of Lévy walk foraging

**DOI:** 10.1371/journal.pcbi.1005774

**Published:** 2017-10-03

**Authors:** Marina E. Wosniack, Marcos C. Santos, Ernesto P. Raposo, Gandhi M. Viswanathan, Marcos G. E. da Luz

**Affiliations:** 1 Departamento de Física, Universidade Federal do Paraná, Curitiba–PR, Brazil; 2 Department of Physics, Universidade Federal do Rio Grande do Norte, Natal–RN, Brazil; 3 Laboratório de Física Teórica e Computacional, Departamento de Física, Universidade Federal de Pernambuco, Recife–PE, Brazil; 4 National Institute of Science and Technology of Complex Systems, Universidade Federal do Rio Grande do Norte, Natal–RN, Brazil; CSIC, SPAIN

## Abstract

We study through a reaction-diffusion algorithm the influence of landscape diversity on the efficiency of search dynamics. Remarkably, the *identical* optimal search strategy arises in a wide variety of environments, provided the target density is sparse and the searcher’s information is restricted to its close vicinity. Our results strongly impact the current debate on the *emergentist* vs. *evolutionary* origins of animal foraging. The inherent character of the optimal solution (i.e., independent on the landscape for the broad scenarios assumed here) suggests an interpretation favoring the *evolutionary* view, as originally implied by the Lévy flight foraging hypothesis. The latter states that, under conditions of scarcity of information and sparse resources, some organisms must have evolved to exploit optimal strategies characterized by heavy-tailed truncated power-law distributions of move lengths. These results strongly suggest that Lévy strategies—and hence the selection pressure for the relevant adaptations—are robust with respect to large changes in habitat. In contrast, the usual emergentist explanation seems not able to explain how very similar Lévy walks can emerge from all the distinct non-Lévy foraging strategies that are needed for the observed large variety of specific environments. We also report that deviations from Lévy can take place in plentiful ecosystems, where locomotion truncation is very frequent due to high encounter rates. So, in this case normal diffusion strategies—performing as effectively as the optimal one—can naturally emerge from Lévy. Our results constitute the strongest theoretical evidence to date supporting the evolutionary origins of experimentally observed Lévy walks.

## Author summary

How organisms improve the search for food, mates, etc., is a key factor to their survival. Mathematically, the best strategy to look for randomly distributed re-visitable resources—under scarce information and sparse conditions—results from Lévy distributions of move lengths (the probability of taking a step *ℓ* is proportional to 1/*ℓ*^2^). Today it is well established that many animal species in different habitats do perform Lévy foraging. This fact has raised a heated debate, viz., the *emergent versus evolutionary* hypotheses. For the former, a Lévy foraging is an emergent property, a consequence of searcher-environment interactions: certain landscapes induce Lévy patterns, but others not. In this view, the optimal strategy depends on the particular habitat. The evolutionary explanation, in contrast, is that Lévy foraging strategies are adaptations that evolved via natural selection. In this article, through simulations we exhaustively analyze the influence of distinct environments on the foraging efficiency. We find that the optimal procedure is the same in all situations, provided density is low and landscape information is scarce. So, the best search strategy is remarkably independent of details. These results constitute the strongest theoretical evidence to date supporting the evolutionary origins of experimentally observed Lévy walks.

## Introduction

How organisms improve—or even optimize—the search efficiency of randomly distributed resource targets is a key question in the study of several biological processes (e.g., foraging and pollination). The search efficiency may even determine matters such as the individuals’ survival and the viability of future generations [1, 2]. The understanding of the rules that guide animal foraging and the unveiling of the main factors leading to successful strategies are crucial topics in behavioral ecology [1–8]. Random search models have been largely employed in the last decades to address these and related issues [1, 2]. In most cases, the quest for an efficient search strategy requires the explicit or implicit definition of a proper choice for the efficiency function *η*, whose maximization constitutes the focus of the *optimal foraging theory* [1, 2, 5–9].

Thanks to the recent advances in the acquisition of huge amounts of empirical data [10–30] and to the great improvement in the statistical methods of data analysis and statistical inference [30–45], it is now settled [10–30] that several species do perform Lévy walk foraging in many circumstances. In other words, the tail of the distribution of their move lengths—while looking for targets such as food, mates, sheltering, territory invaders, etc.—is well described by a truncated power-law function.

The earliest suggestions that Lévy processes could be related to the movement of organisms, based on empirical studies, date back to the late 1980’s [46–48]. In the 1990’s the first theoretical framework to explain this evidence appeared in the form of the so-called Lévy flight foraging hypothesis [1, 49]: Organisms must have evolved via natural selection to exploit search strategies that optimize the search efficiency provided by Lévy distributions of move lengths under scarce information and sparse targets conditions (for a detailed discussion, see, e.g., [50]).

Since in several contexts the existence of Lévy foraging has ceased to be a matter of debate, other fundamental questions have progressively come into the spotlight [50–60] (but see [61]). For example, under precisely which circumstances is Lévy flight foraging advantageous over Brownian searches (e.g. described by exponential or gamma distributed move lengths) is still an ongoing issue. Another central question concerns the underlying mechanism of Lévy foraging as having *emergent* rather than *evolutionary* origins [50, 51]. According to the former, a Lévy foraging pattern should arise as an emergent property, i.e. a side-effect of complex interactions between the searcher and the environment. The evolutionary explanation, in contrast, is that Lévy foraging strategies are adaptations that evolved via natural selection.

This matter remains controversial as recent results have claimed support for both emergentist [14, 20, 21, 32, 62–68] and evolutionary [12, 16, 33, 69–76] views. As already emphasized, optimal or even suboptimal search outcomes are essential to guarantee the endurance of an individual or a species. Therefore, the question about the emergent versus evolutionary origin of the empirically observed Lévy foraging behavior becomes crucial.

Here we address this issue by investigating—through random search ideas and a (very general) reaction-diffusion model—the influence of distinct landscapes on the optimal foraging strategy. The searcher’s degree of diffusivity is controlled by the power-law exponent *μ* of the distribution of step lengths, *P*(*ℓ*) ∼ *ℓ*^−*μ*^. Hence, ballistic, superdiffusive, or diffusive search strategies correspond to *μ* → 1, 1 < *μ* < 3, or *μ* ≥ 3, respectively [1]. The reactive component is represented by the detection and consumption of target sites once they enter the searcher’s perceptive range.

We perform an extensive analysis of the search efficiency in a great diversity of heterogeneous landscapes, namely: (i) nonuniform patches containing homogeneous distributions of targets; (ii) targets distributed fractality as a Lévy dust; and (iii) Lévy dusts of both patches and inner targets. A large collection of assorted environments is thus studied by varying the density of targets, number and size of patches, degree of heterogeneity and fragmentation, and fractal dimension of the Lévy dusts (including the homogeneous distribution of targets as a relevant limit case).

The *emergentist* view, relying mostly on the presence of complex searcher-targets interactions, should be sensitive (and respond accordingly) to the specificities of the landscape. Therefore, diverse optimal search strategies should arise in rather distinct environments: the best search ‘protocol’ would depend on the particular habitat, does not existing an universal (good in all instances) behavior. In contrast, our main result is that the optimal search strategy is essentially *the same* in all situations considered, provided the targets density is sparse and environment information is scarce. In other words, as long as targets are not often found within the searcher’s perceptive range and leaving the present spot to look for resources remains a major activity, the general properties of the best search strategy appear to be remarkably independent of landscape details.

This basically “insensitive” character of the optimal strategy then suggests the interpretation favoring the *evolutionary* view, which concurs with the Lévy flight foraging hypothesis. Indeed, as we also report, deviations from this behavior are shown to be relevant in plentiful environments, in which the importance of extensive searching is reduced. Nevertheless, an alternative (though less probable) scenario supporting the emergentist view would be that in which essentially the same optimal Lévy foraging strategy arises in rather diverse landscapes, even considering the distinct specificities of the searcher-environment interactions (or trade-offs) in each particular case.

Such questions have led to a lively debate in the literature. We cite here only 3 such examples.

1. In an article provocatively titled “Ultimate failure of the Lévy Foraging Hypothesis”, Benhamou and Collet have argued that composite Brownian walks consisting of two scales are better than Lévy walks [77]. However, scale-specific strategies presupposes that the foragers have some information about the target distribution. Thus, the direct comparison with clueless Lévy walks should be viewed with a certain reservation. On the other hand, they claim correctly that natural selection should favor the evolution of search strategies for which environmental feedback is possible, but conclude that “strange” scale-free kinetics are somehow ruled out without giving plausible reasons for so. We disagree that scale-free kinetics cannot have evolved and as evidence cite the recent work of Gutiérrez and Cabrera involving a conductance-based neural coding scheme that reproduces foraging trajectories [78]. Moreover, for searchers with high cognitive skills see [79, 80]. In any case, the results we report below show that environmental feedback is most likely present in a wide variety of landscapes, still, organisms get rewarded for choosing generalistic optimal or near-optimal strategies.
2. Pyke, in an article subtitled “It’s time to abandon the Lévy foraging hypothesis”, criticizes many aspects of the hypothesis [51]. A rebuttal of all the criticisms is beyond the scope of the present work. We merely point out here that if the evolutionary view discussed above is even partially correct, it may be premature to abandon the hypothesis. The results we report next strongly support the evolutionary view.
3. Finally, we mention the recent work of Reynolds, who has argued that constraining the research on Lévy walks to the confines of optimal foraging theory can be restrictive [60]. This is a valid point. We have argued in [50] that the complete answer may involve a combination of both the evolutionary and the emergentist views.

We organize this work as follows. First, we present a brief description of certain relevant aspects associated to the foraging behavior under the emergent and evolutionary points of view. Second, we build the diverse heterogeneous landscapes in which the searches take place. And third, we define the general random search model to be employed. Then, the results for the efficiency of search strategies in each of these landscapes are discussed and their connection with the issue of emergent vs. evolutionary foraging behavior is established. Finally, ending remarks and conclusions are drawn.

### Factors associated to the origin of efficient foraging behavior

#### Emergence and evolutionary pressure

Perhaps, the most fundamental question regarding efficiency in foraging—or more generally in biological encounters (as discussed, e.g., in [81, 82])—is the real necessity for optimal strategies. Animals can display complex and rich habits, associated to the multiple tasks they must accomplish. Foraging is certainly one of them. But to develop proper characteristics (metabolical, morphological, cognitive, ecological, etc) allowing an effective random search strategy should be the case only if foraging becomes a bottleneck for survival.

Evolution through natural selection is probably the simplest yet most powerful universal mechanism driving the huge diversity and great adaptability of species on all scales, from isolated groups of species in small islands to metapopulations in biomes. Generally, selective pressure does not act (or acts only mildly) on a certain feature if it is irrelevant for the fitness of the individuals. In this context, a particular trait, say a specific foraging behavior, may be a side-effect of another evolutionary process, as proposed in [60]. Or it may just be an emergent trade-off, a direct response to the environment. But note that emergent behavior does not imperatively lead to advantageous strategies [83, 84]. This can be the case, although only fortuitously by coincidence [85, 86].

A point barely discussed in the foraging literature, however greatly emphasized in population dynamics (see, e.g. [87]), concerns the type of patterns arising in terms of the individuals responses to the environment. They are distinct if such responses are either local or global. In fact, according to the so called Moran effect [88, 89]—related to the habitat as a whole (a rather relevant aspect when the random search takes place in a reasonable fraction of it)—an important ingredient to induce emergence is the build-up of an awareness by the forager of long-range correlations existing in the landscape. Only immediate and local responses to stochastic stimuli imposes serious restrictions for the rise of a full extended environment-related behavior [90], of the kind conceived as an alternative to the evolutionary view [32, 62–68].

The above considerations bring an extra ingredient into the discussion. Either (i) encounter rate optimization is not needed or else (ii) it is needed, leading to selection pressures. We consider both cases below.

Suppose first there is no pressure for higher biological encounter rates. As already observed, then for a “brimming” emergent behavior an individual must possess certain inherent skills (like long-term memory and/or specific long-range detection power) to perceive territorial variations on a large enough scale. In some instances, the correct abilities might have evolved due to unrelated evolutionary forces, i.e. a matter of “lucky” coincidence [60]. But in others, such abilities may not be useful at all in a given landscape. Thus such abilities may not arise in the first place—hence may not bolster emergent Lévy foraging. In this case, emergence in the Lévy foraging context would not be ubiquitous.

On the contrary, if optimization is needed, the innate emergent behavior in a certain ecosystem can arise only if it corresponds to a good foraging strategy. In each distinct landscape, the necessary “proficiency” to identify the habitats’ long-range structures might be different, e.g., to spot spatial correlations of resources in the African savanna certainly requires features and traits different from those necessary to do the same in the Amazon rainforest. Pure emergence leading to optimal strategies thus demands the extra-ordinary coincidence of fortuitous matches between habitats and just the right kinds of traits. Such coincidences would be analogous to demanding eyesight to have fortuitously “emerged” independently in different mammals living in widely differing habitats as a side-effect of distinct unrelated adaptations, rather than having evolved as an adaptation via natural selection. Given the huge range of situations, although such lucky coincidences can take place, they must be extremely rare.

#### The evolutionary robustness of foraging strategies

Macroevolution displays a rather interesting dynamics, where bursts of high evolutionary variations and extinctions (hectic phase) are followed by long periods of metastable configurations (stasis phase). This intermittency, which has become known as punctuated equilibrium [91], is well established from the analysis of fossil records [92–94]. Regardless the basic reasons for such a pattern [95], it strongly contrasts with what should be expected from the natural selection taking place at the much shorter scales of several generations [96, 97]. Hence, pathways of normal evolution—allowing gradual adjustments to a varying habitat—might represent a somehow limited resilience mechanism if considered at long times scales [92, 93]. Indeed, depending on the magnitude and rate of the changes in the ecosystem (e.g., with the potential of strongly unbalance the trophic interactions [98]) incremental skills acquired through natural selection could not suffice to preclude intensive extinction [99].

Among distinct evolutionary behaviors, however, one can think about an hierarchical order [50], with the existence of those granting a high power of adaptability [100]. These features, which are more plastic in the sense of being more robust to distinct environmental configurations, once incorporated into the individuals habits are likely do not suffer subsequent drastic alterations [101]. For instance, specific characteristics of shorebirds are strongly related to foraging behavior as it concerns the type of food they need to detect and handle [102], e.g. the case of bill length determined by the type of hunting, either visual or tactile [103]. On the other hand, certain general morphologic aspects (as the hindlimb structure) does not seem to have affected along the time the foraging movement strategies of shorebirds [103].

A particularly intriguing evolutionary history is that of the neornithine. This whole crown group is believed to have fairly similar behaviors of the modern birds [104] since their extensive diversification in the late Cretaceous [105]. So, they have gone through the huge extinctions of the K/Pg boundary probably maintaining many of their ecological traits. It indicates strong endurance of their original behaviors [101]. In fact, Lévy foraging strategies are common among Aves [16, 25, 29, 106], a class in which flexible and generalist foraging strategies are not unusual, as exemplified by frugivorous birds [106].

It is worth mentioning that trace fossils dating fifty million years ago [22] have given support to such idea associating Lévy walks to the origin of optimal search behavior. Furthermore, present day communities still maintaining the hunter-gatherer tradition may constitute a nice window into the human past evolution. So, the fact that the Ju/’hoansi of Botswana and Namibia [107] and the Hadza of Tanzania [108] follow Lévy walk foraging patterns in their activities is another evidence for the Lévy hypothesis.

#### Important features of efficient foraging strategies, Lévy walks and other alternative search behaviors

There are situations where the specific foraging behavior is irrelevant [109]. In essence, either because energetic resources are plentiful or when enough information, cognitive power and strong detection skills preclude the necessity of a complete random search. In contrast, in (a) clueless scenarios and (b) scarce conditions [1, 50], the particular features of searching may constitute the difference between surviving or perishing [110]. In these cases, probably the most important characteristics that any foraging “protocol” should possess are superdiffusion and scale invariance (see, e.g., [9, 75])—incidentally, two inherent properties of unrestricted Lévy strategies [1].

At low densities, in fact, typical Brownian-like motion (leading to normal diffusion) cannot account for an extensive territory exploration, necessary to find enough quantities of targets (e.g, resources). At the same token, for no information about eventual targets distribution patterns (defining typical spatial scales in the environment) or if the resources dynamics is sufficiently fast (not allowing specific targets spatial arrangements), then a foraging behavior too focused on specific spatial scales can be a disadvantage. Hence, a better (eventually optimal) solution would be to adopt scale invariant strategies.

These properties, especially superdiffusion, can be obtained from a variety of random walks, but there are important technical limitations. For instance, correlated random walks (CRWs)—whose step lengths have well defined mean and variance and the turning angles are (Markovian) correlated, so with a certain directional bias—can display superdiffusion, but up to a limiting time *τ* [111]. Actually, there are concrete conditions determining up to which *τ* a given random walk can maintain superdiffusion [44]. If the biological relevant time *T* involved in the foraging is longer than *τ*, then such strategy (considering (a) and (b) above) may be worthless. Remarkably, truncated Lévy walks, more commonly used as models for animal foraging [1], do present *τ*’s which are extremely long [112]. Thus, they are able to hold supperdifusion in many long foraging contexts [113, 114]. Furthermore, for large time scales the value of *T* can suffer important fluctuations. So, a robust and lasting strategy should have huge *τ*’s, the case of truncated Lévy walks (for usual Lévy walks *τ* is in fact infinite).

In a landmark work [115], it has been shown how certain kinds of Lévy flights could be written in terms of nondifferentiable infinite series, known as the Weierstrass function. This fundamental technical result (further explored in different instances, e.g, [116–118]) gives the theoretical support for a relevant proposal of composite random walks (CompRWs) in ecology (cf. [32, 119, 120]).

For a brief contextualization, let us assume the non-truncated case in 1D (a general mathematical analysis comparing CompRW with truncated and non-truncated Lévy walks will be the subject of a future contribution). In general terms, for a step length probability density function given by (for *ℓ*_0_ ≤ *ℓ* < ∞, with *ℓ*_0_ a lower cut-off: there is no meaning to take random steps smaller than a given distance *ℓ*_0_, related to an individual biological characteristics) [118, 121]

$$\begin{array}{c} P_{comp}\left(\ell \right)=\{\begin{array}{c} \sum_{n=1}^{n=N}\left(w_{n}/\ell_{n}\right)\;\exp\left[-\left(\ell -\ell_{0}\right)/\ell_{n}\right],\;\ell_{0}\leq \ell ,\\ 0,\;\text{otherwise}. \end{array} \end{array}$$ (1)

The normalization imposes that ∑_*n*_ *w*_*n*_ = 1. Here the *ℓ*_*n*_’s are typical length scales and the *w*_*n*_’s the corresponding weights of the *P*_*comp*_ “modes” *n*. Observe, therefore, that we have 2*N* free parameters: the *N* lengths *ℓ*_*n*_, *N* − 1 weights *w*_*n*_ (recall the normalization condition) and *N* itself. For Eq (1), the shifted first (second) moment of *P*_*comp*_, given the average step length (a quantity related to the step lengths variance) reads

$$\begin{array}{ccc} \left\langle \ell -\ell_{0}\right\rangle & = & \int_{\ell_{0}}^{\infty }\left(\ell -\ell_{0}\right)\;P_{comp}\left(\ell \right)\;d\ell =\sum_{n=1}^{n=N}w_{n}\ell_{n},\\ \left\langle {\left(\ell -\ell_{0}\right)}^{2}\right\rangle & = & \int_{\ell_{0}}^{\infty }{\left(\ell -\ell_{0}\right)}^{2}\;P_{comp}\left(\ell \right)\;d\ell =2\sum_{n=1}^{n=N}w_{n}\ell_{n}^{2}. \end{array}$$ (2)

By the central limit theorem, any distribution having finite first and second moments will converge to normal diffusion after a certain number of steps determined by *τ*. Thus, from the realistic assumption that all *ℓ*_*n*_’s are finite, we can have true superdiffusion (i.e., for any time scale) only for an infinite number of modes. In this way, rigorously CompRWs can describe Lévy walks only in such *N* → ∞ limit. Nonetheless, it has been shown [121] that *N* does not need to be large for *P*_*comp*_ to resemble power-law distributions (so Lévy-like), describing superdiffusive foraging for relatively long time scales.

Many foraging models, and Lévy walks are not an exception, assume that the searching is the only activity being performed by the individual. But surely ecological processes are not exclusive: while looking for targets, an animal may be forced to change the behavior for a while, say, when facing some unexpected event. But this does not mean that optimal foraging could not be pursued in multi-task assignments. For instance, a detailed study has shown that collective searching [122]—in which constraints, such as avoidance of group dispersal, are imposed—still can be profit from Lévy strategies, but then certain spatial correlations must be imposed. In such context, CompRW should be a rather interesting alternative [19, 121].

An instructive example is that of mussels movement, associated to involving individuals interactions (including both cooperation and competition). During intertidal flats, they arrange themselves in regularly spaced clumps [14]. In such complex environment with strong feedback (thus, far more sophisticated than the usually considered foraging scenario [1, 50]), it has been reported [14] that the locomotion shaping the patterned beds are well described by Lévy walks. Nevertheless, in a reexamination of the original experimental data, CompRWs seems to yield better fittings [43]. This should not be a great surprise; in fact, it illustrates that pure Lévy ‘motifs’ can be partially altered due to the group interplay [122]. Important here is that the difference between the truncated Lévy walk and CompRW observed in the fittings (Fig 1 (a) in [43]) would not lead to a significant difference between the resulting search efficiencies (see, e.g., a quantitative analysis of local correlation effects in Lévy searching outcomes in [123]). For the results of Fig 1 (b) in [43], the discrepancy is much more pronounced. However, in this case a larger data set is used. It includes many step lengths that are rather small (e.g., typically the mussels are 7 mm long and *ℓ*_0_ is set around 0.02 mm), specially considering an aquatic environment, a media that can induce involuntary movement. Hence, such a data may be considered with a certain care for the study of foraging efficiency.

The previous discussions bring about distinctions between Lévy walks and CompRWs in terms of random search optimization (but relevant to mention that selection pressures could render CompRWs to display certain characteristics of Lévy walks [124]). The fact is that to a great extent a foraging behavior taking fully advantage of the explicit form of *P*_*comp*_ would be much more complex than Lévy strategies. Indeed, for an appropriate number of modes *N*, eventually an individual would be able to have a strategy incorporating the most important length scales in its habitat as well as the relative frequency of these scales (given by the *w*_*n*_’s). But then, built-in biological mechanisms should be available so that all the environment specificities could be identified. These mechanisms should be particularly refined in the case of changing environments, when the sets of *w*_*n*_’s and *ℓ*_*n*_’s and even *N* are dynamical parameters—exactly the type of situation posing strong evolutionary challenges. Moreover, certain skills (like non-directional sensory cues [125]) are necessary to allow proper switchings between the modes of movement behavior. In this way, evolutionary CompRWs might represent a later stage to Lévy walks, with many hierarchical ecological processes successively forging the species full behavior through intermediate states.

Surely, it is plausible that some evolutionary paths would bring directly to composite random walks. However, the generality, independence on landscape details, extremely economical in terms of free parameters and ecological plasticity [126] makes Lévy walks the natural candidate in testing evolutionary versus emergent origin of optimal foraging.

#### The framework adopted here and some previous discussions in the literature

It becomes clear from the above the difficulty in establishing the genesis of efficient random search strategies. In fact, we share similar views of many others: that it is unlikely that an unique explanation can account for every imaginable scenario. However, solid and general trends in movement ecology may point to a common (although not universal) mechanism determining the origin of optimal foraging behavior in a large number of cases.

In this context, our starting point, or framework, is the Lévy flight foraging hypothesis (LFFH). We will demonstrate in a systematic way in this contribution that Lévy walks—in the form of an asymptotic power-law distribution of step lengths, with a single relevant parameter *μ* (see next section)—does lead to optimal searching efficiency in a remarkably broad range of landscapes. This fact shows clearly that Lévy walks are robust and adaptively plastic. This is distinct from emergence, where conceivably specific-environment driven forces would yield different kinds of foraging behavior.

Admittedly, dedicated strategies (DS) with fine tuned parameters (like CompRWs) may be able to provide good foraging efficiencies, but at a higher cost in an evolutionary sense. In fact, the proper traits required by DS may need more time and higher selection pressure to evolve. On the other hand, the biologically much simpler Lévy strategies (LS) (not demanding switching or long range sensorial clues) could go unchanged during habitat transformations. Thus, since LS are cheaper, throughout the evolutionary history one could expect, when the case, LS evolving to DS (LS → DS), but DS → LS would be less probable. Also, once the LS becomes an adaptation, the resulting efficiency might relieve the evolutionary pressure towards even better strategies. So, the commonly observed LS could be a relic or descendant of very ancient adaptations [22, 107, 108], whereas DS could have evolved from more recent processes. These views are in accordance with the ideas in [60] and also with a hierarchical LFFH proposal in [50].

Finally, we stress that the origins of optimality in Lévy searches is an old question [49] (e.g., surveyed in [1]). For more recent discussions, we observe the following. In [75] simulations in different types of environments have addressed how the intrinsic exploitation/exploration trade-offs of Lévy walks can lead, under certain conditions, to a higher efficiency than other models. In our work the focus is different. We show that an intrinsic Lévy walk behavior can provide overall better performance in diverse landscape structural geometries and argue that such plastic behavior, favoring adaptation, should have evolutionary origins. In the search for possible mechanisms yielding the high efficiencies of Lévy walks, it has been proposed that an effective intermittency—arising from singular temporal distribution of reorientation events—may be key for the optimal strategy [127]. This is in sound agreement with our analysis in the super-dense landscape (see the section: The super-dense limit): even when there is no need for a fine-tuned strategy, optimal Lévy walks with *μ* ∼ 2 result in slightly higher efficiency. Further, the possible mechanisms underlying Lévy walks have received great attention. For instance, swarm dynamics [128], biological simulated annealing [129], and even chaotic neuronal dynamics [28], have been pointed as potential candidates generating Lévy dynamics. Our present findings seem to indicate that all such processes could be incorporated as adaptations through natural selection.

## Materials and methods

### Search landscapes

Patchy landscapes [130–138], including some hierarchical structures consistent with scale-free fractal patterns [10, 139–144], are plentiful in nature. To model such environments, we consider a number of heterogeneous distributions of point-like targets placed in a two-dimensional squared space of side length *M* = 10^4^ and periodic boundary conditions. The use of periodic boundary conditions leads to the same qualitative results as those of random searches in large spaces with limiting borders, but with the technical advantage of improving the statistical analysis. Here we are mostly interested in the study of the impact of the environments diversity on search dynamics under sparse conditions. We describe below the landscapes analyzed in this work. But also some discussion about the targets features is in order.

### Heterogeneous patches with Euclidean distribution of targets

**Fig 1:**
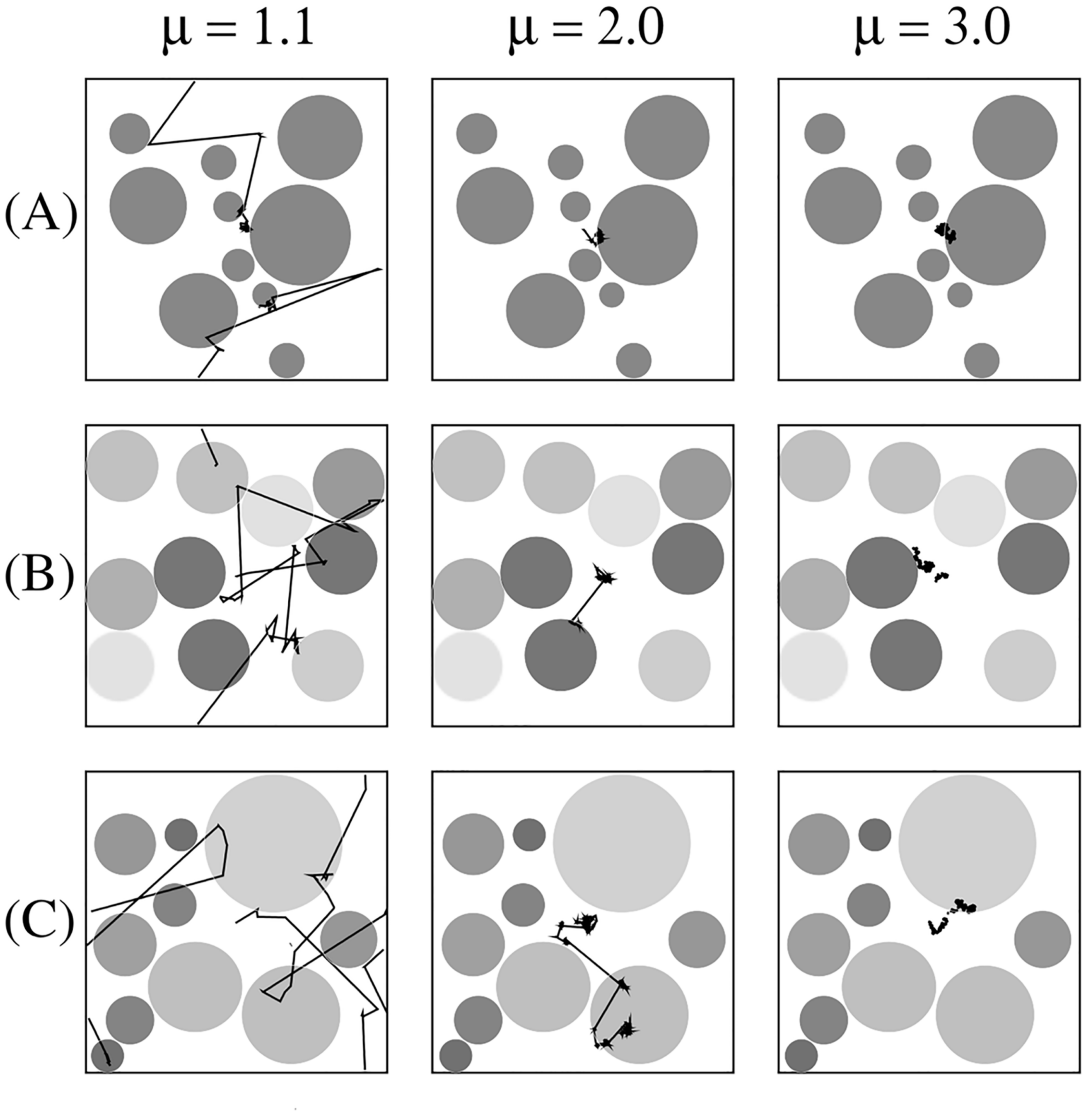
Heterogeneous search landscapes with representative trajectories of different strategies. Fragmented search landscapes containing *N*_*t*_ = 10^4^ targets placed in *N*_*p*_ = 10 heterogeneous patches (gray regions) with: (A) same average distance between inner targets, $l_{t}^{\left(p\right)}=100$, and radii uniformly distributed in the range 0.03*M* ≤ *R*^(*p*)^ ≤ 0.3*M*, *M* = 10^4^; (B) same radius, *R*^(*p*)^ = 0.1*M*, and $l_{t}^{\left(p\right)}$ uniformly distributed in the range $5\leq l_{t}^{\left(p\right)}\leq 350$; and (C) distinct sizes uniformly distributed in the range 0.03*M* ≤ *R*^(*p*)^ ≤ 0.3*M*, but fixed number (10^3^) of inner targets per patch, so that $17\leq l_{t}^{\left(p\right)}\leq 170$. The darker the patch, the higher its homogeneous density of inner targets. We also show typical paths of a searcher with power-law (Lévy-like) distributions of step lengths displaying different degrees of diffusivity: nearly ballistic (*μ* = 1.1), superdiffusive (*μ* = 2.0), and Brownian (*μ* = 3.0). In this illustrative example the search ends upon the finding of only 10 targets.

We start by defining landscapes characterized by the presence of *N*_*p*_ circular patches indexed by *p* = 1, 2, …, *N*_*p*_, with radius *R*^(*p*)^ and average distance $l_{t}^{\left(p\right)}$ between homogeneously (Euclidean) distributed inner targets. The total number of targets is *N*_*t*_ = 10^4^ and the targets density in the *p*th patch is inversely proportional to ${\left[l_{t}^{\left(p\right)}\right]}^{2}$. We mention that the statistical analysis of searches in these heterogeneous landscapes, as well as in the uniform case of patches with same *R*^(*p*)^ and $l_{t}^{\left(p\right)}$, has been partially performed in [81, 145]. Three types of configurations are considered:

1. Random sizes: patches with fixed $l_{t}^{\left(p\right)}=100$ and radii uniformly distributed in the interval 0.03*M* ≤ *R*^(*p*)^ ≤ 0.3*M*, resulting in a distribution of numbers of targets per patch [see Fig 1(A) with *N*_*p*_ = 10 patches];
2. Random targets densities: patches with fixed *R*^(*p*)^ = 0.1*M* and targets densities uniformly distributed in the interval $5\leq l_{t}^{\left(p\right)}\leq 350$, also presenting a distribution of numbers of targets per patch [Fig 1(B)]; and
3. Random sizes and targets densities: targets equally distributed in the patches with different radii, 0.03*M* ≤ *R*^(*p*)^ ≤ 0.3*M*, thus generating lower (higher) inner densities in larger (smaller) patches, with $17\leq l_{t}^{\left(p\right)}\leq 170$ [Fig 1(C)].

As we set the searcher’s perceptive range to unity, we consider that values $l_{t}^{\left(p\right)}∼10$ and ∼ 10^2^ can be respectively assigned to locally dense and sparse targets distributions. In each case above, we have also analyzed less fragmented landscapes with a smaller number *N*_*p*_ = 5 of denser patches (see Results below).

### Lévy dust distribution of targets

**Fig 2:**
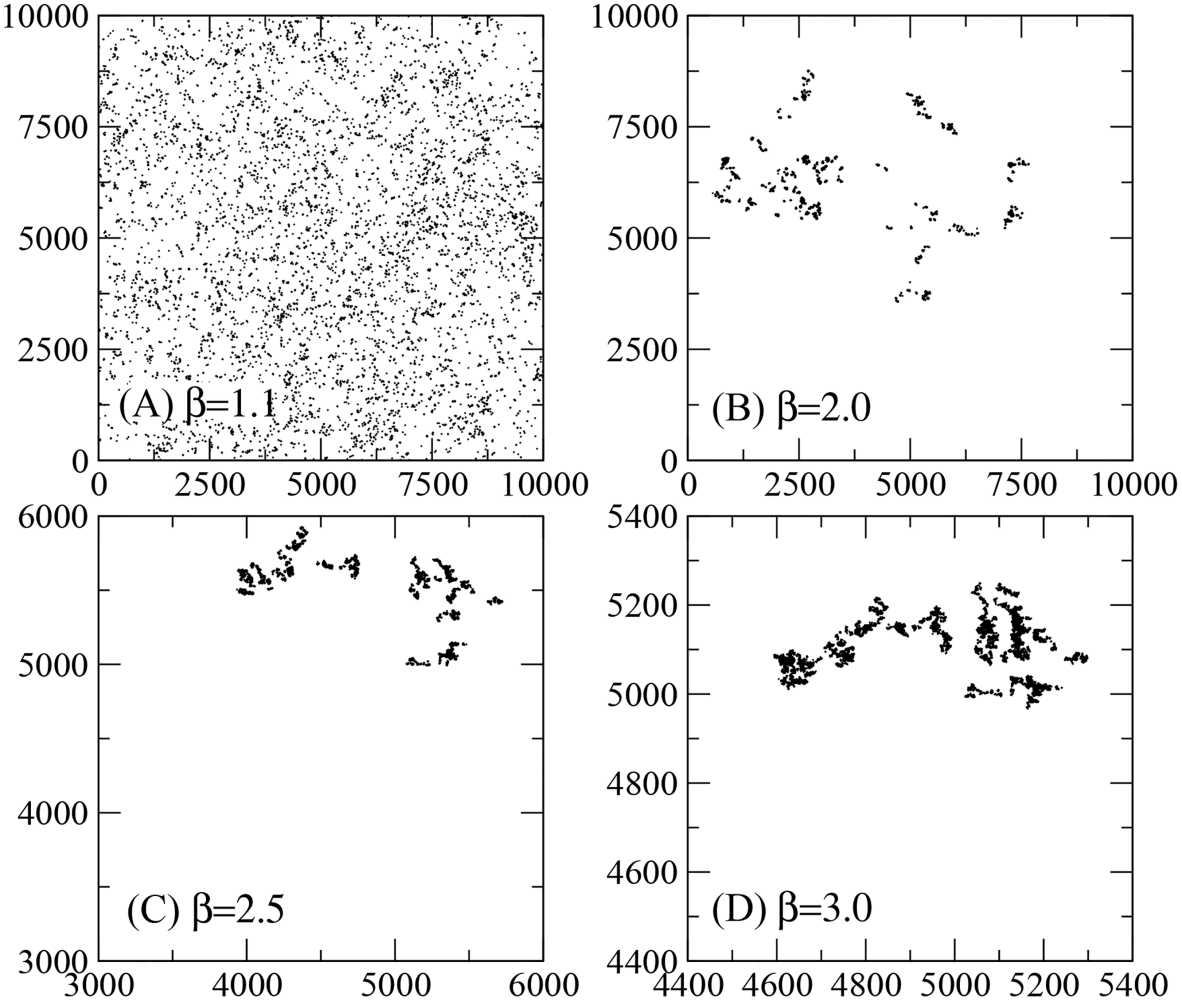
Lévy dust distribution of targets. Search landscapes containing Lévy dust distributions of *N*_*t*_ = 10^4^ targets (see main text), drawn from Eq (1) with *d*_0_ = 1, *d*_max_ = *M* = 10^4^, and (A) *β* = 1.1, (B) *β* = 2.0, (C) *β* = 2.5, and (D) *β* = 3.0. Larger values of *β* increase the degree of clustering of targets. The bouncing of coordinates technique applied to the *β* = 1.1 case results in a nearly homogeneous targets distribution.

We also considered a distribution of *N*_*t*_ = 10^4^ targets whose positions form a so-called *Lévy dust*. In this case, the targets locations correspond [46, 146, 147] to the points visited by a Lévy walker (not to be confounded with the searcher itself) following the probability density function (pdf) of move sizes *d* given by

$$\begin{array}{c} P_{t}\left(d\right)=\{\begin{array}{c} 0,\;\;\;d<d_{0}\;\text{or}\;d>d_{\text{max}},\\ A_{t}d^{-\beta },\;d_{0}\leq d\leq d_{\text{max}}, \end{array} \end{array}$$ (3)

and homogeneous distribution of turning angles in the interval [0, 2*π*). Above, the parameter *d*_0_ = 1 (*d*_max_ = *M*) represents the smallest (largest) move allowed, and *A*_*t*_ is the normalization constant. In the large-*N*_*t*_ limit, the set of points of the Lévy dust has scale-free properties within the interval (*d*_0_, *d*_max_), and fractal dimension *d*_*f*_ = *β* − 1 [46] (we observe that for actual environments with fractal distributed resources the reader can see, e.g., [148, 149]). The *β* = 3 Brownian limit corresponds to the Euclidean aggregation of targets in two-dimensional space. Some examples are illustrated in Fig 2. Notice that larger values of *β* imply denser clusterings of targets occupying a relatively small fraction of the search space [see, e.g., Fig 2(D)]. On the other hand, a nearly homogeneous landscape results from the case *β* = 1.1, as seen in Fig 2(A). This feature allows an interesting comparison with the random searches performed in homogeneous environments, the earliest scenarios studied in the context of Lévy foraging [49].

We observe that, due to the finite landscape, border effects should be carefully considered along the construction of the Lévy dust. Indeed, the frequency of ultra-long steps increases as *β* approaches unity. The undesirable situation in which the end coordinates of a given move eventually reach a region beyond the borders of the environment must be fixed. In order to do it, we have applied the bouncing of coordinates technique [147].

### Lévy dust distributions of patches and inner targets

The previous types of landscapes can be combined to create a great diversity of environments with heterogeneous (Lévy dust) distributions of both patches and targets.

**Fig 3:**
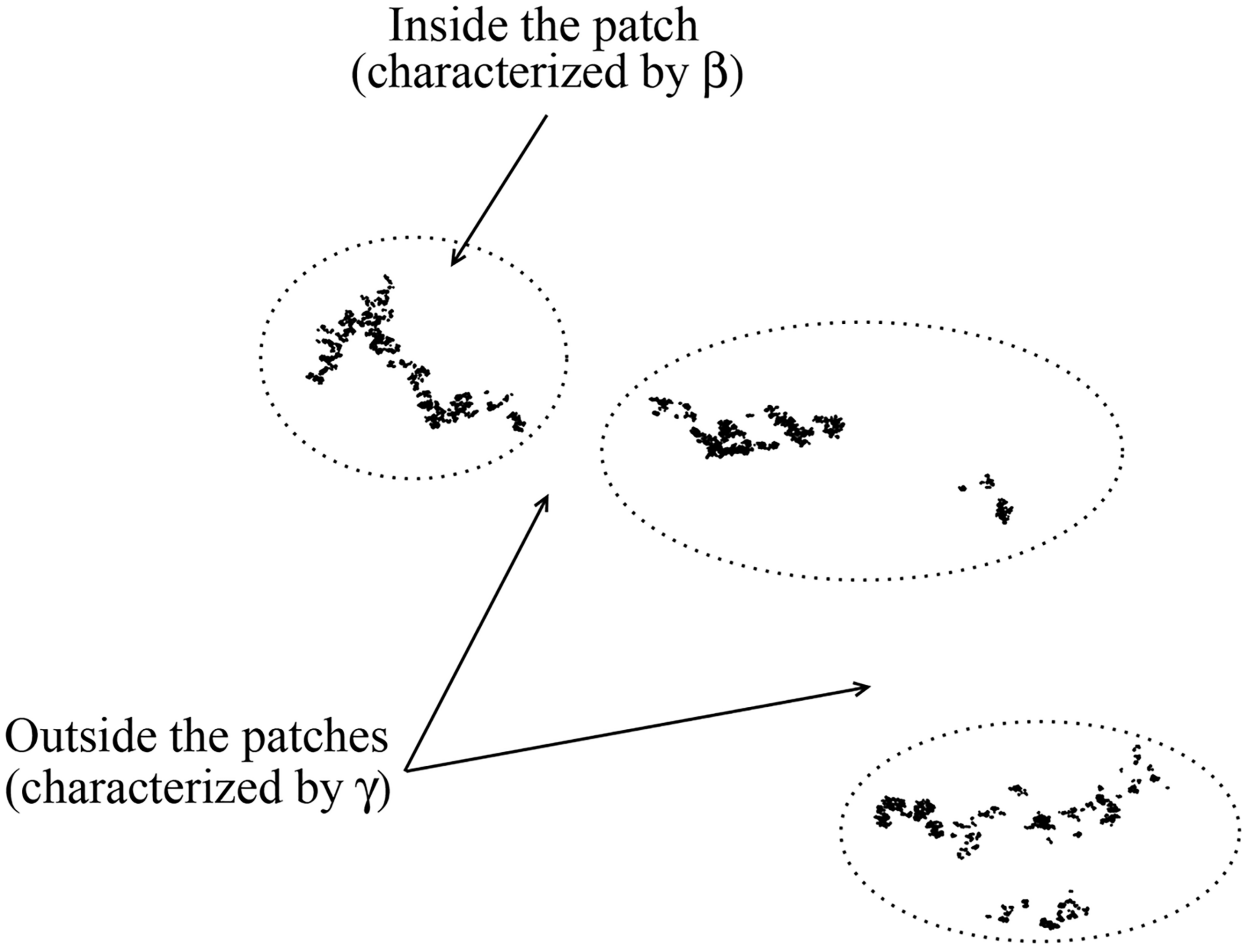
Construction of a fractal patch environment. Illustration of a search landscape with *N*_*p*_ = 3 patches and *N*_*t*_ = 15000 targets (5000 targets per patch), forming Lévy dust distributions (see main text). Here, *β* = 2.5, *d*_0_ = 2, *d*_max_ = *M*/10 in Eq (1), and *γ* = 2.0, *r*_0_ = 500, *r*_max_ = *M* = 10^4^ in Eq (2). The parameters are chosen so that the patches do not overlap. Dotted lines are only a guide to visually delimit the patches regions.

We now place *N*_*t*_ targets equitably among *N*_*p*_ patches. Inside each patch, the targets are distributed as a Lévy dust of exponent *β*, as in Eq (3). Moreover, the patches “seeds” (or centers) also form a Lévy dust characterized by another exponent *γ*, i.e. the distances *r* between the patches seeds are drawn from the pdf

$$\begin{array}{c} P_{p}\left(r\right)=\{\begin{array}{c} 0,\;\;\;r<r_{0}\;\text{or}\;r>r_{\text{max}},\\ A_{p}\;r^{-\gamma },\;r_{0}\leq r\leq r_{\text{max}}, \end{array} \end{array}$$ (4)

where *A*_*p*_ denotes the normalization constant. A careful choice of parameters is necessary in order to avoid overlap of patches: the inferior limit *r*_0_ should be larger than the typical size of the patch, whereas the superior limit *r*_max_ should be naturally smaller than the environment length. An example is shown in Fig 3 for *N*_*p*_ = 3 and *N*_*t*_ = 15000 (5000 targets per patch), with distributions of patches and targets, respectively, set by *γ* = 2.0, *r*_0_ = 500 and *r*_max_ = *M*, and *β* = 2.5, *d*_0_ = 2 and *d*_max_ = *M*/10. In this case, it no longer makes sense to define the radius *R*^(*p*)^ of a patch, since the parameter *β* governs the degree of clustering of the targets distribution within each patch (the dotted lines in Fig 3 are only a visual guide).

**Fig 4:**
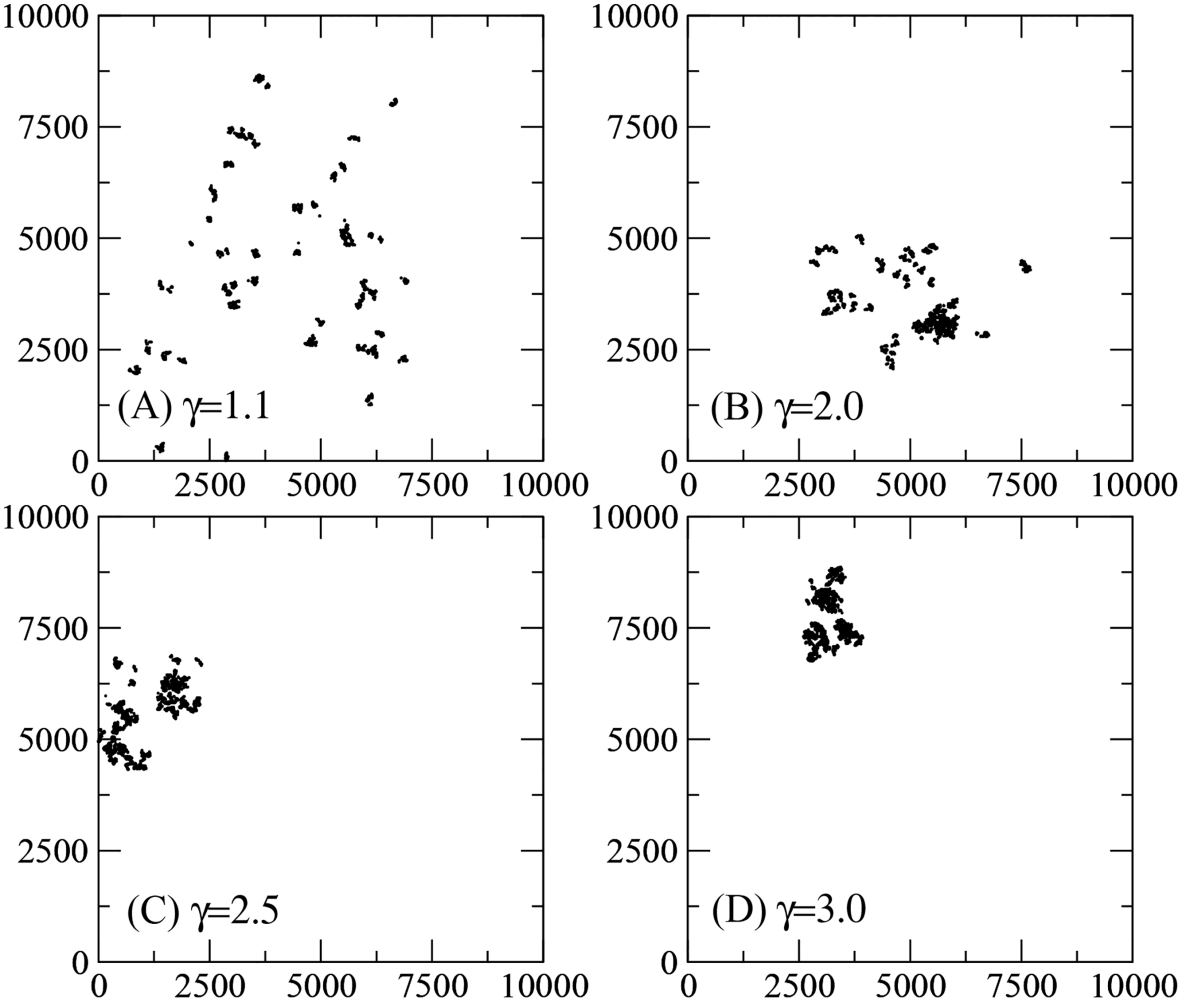
Fractal patches obtained by combining two Lévy dust distributions. Search landscapes containing Lévy dust distributions located in *N*_*p*_ = 50 patches. Here, *N*_*t*_ = 50000 (1000 targets per patch), *β* = 3.0, *d*_0_ = 2, *d*_max_ = *M*/10 in Eq (1), and *r*_0_ = 100, *r*_max_ = *M* = 10^4^, (A) *γ* = 1.1, (B) *γ* = 2.0, (C) *γ* = 2.5, (D) *γ* = 3.0, in Eq (2). For large *γ* the patches are so close that one cannot distinguish them only by visual inspection.

In Fig 4 *N*_*p*_ = 50 patches are depicted (this time no dotted lines helping to delimit the patches are displayed), with a total of *N*_*t*_ = 50000 targets (1000 targets per patch) distributed according to *β* = 3.0, *d*_0_ = 2 and *d*_max_ = *M*/10. Four arrangements of Lévy dusts of patches are shown, with *r*_0_ = 500, *d*_max_ = *M*, and *γ* = 1.1, 2.0, 2.5, 3.0. Notice that a value of *γ* close to unity gives rise to a more widespread distribution of patches, occupying a larger area of the landscape. In contrast, for *γ* = 3 the patches are so close that one cannot distinguish them only by visual inspection. In this case, a large empty space surrounds the region rich in targets.

### Search dynamics and efficiency

Once the several search landscapes have been defined, we next consider the random search model used to evaluate the performance of distinct search strategies.

The searcher starts from a random position in the depleted region outside the patches. The step lengths *ℓ* are taken from the truncated power-law (Lévy-like) pdf

$$P\left(l\right)=\{\begin{array}{ll} 0, & l<l_{0}\;\text{or}\;l>l_{\text{max}},\\ Bl^{-\mu }, & l_{0}\leq l\leq l_{\text{max}}, \end{array}$$ (5)

with *B* denoting the normalization constant, and the turning angles are homogeneously distributed in the interval [0, 2*π*). The searcher can detect a target within a unit perceptive range *r*_*v*_ = 1, with unlimited visits allowed to the targets (but see the next Section). This regime has been termed non-destructive in the literature [1, 49]. The step is truncated if the searcher encounters a target within its perceptive range *r*_*v*_ before traversing the distance *ℓ*. We choose *ℓ*_0_ = *r*_*v*_, since search steps shorter than the perceptive range are meaningless, and *ℓ*_max_ = *M* corresponding to the limit scale of the environment. In fact, it is clear that unbounded (infinite) displacements are naturally forbidden in realistic searches.

Eq (5) with *ℓ*_max_ → ∞ corresponds to the asymptotic large-*ℓ* limit of Lévy *α*-stable distributions governed by the generalized central limit theorem (CLT), with Lévy index 0 < *α* = *μ* − 1 ≤ 2 [1, 2]. Extremely ballistic and superdiffusive random walk dynamics are represented by *μ* → 1 and 1 < *μ* < 3, respectively. In contrast, values *μ* ≥ 3 lead to the *α* = 2 Brownian (diffusive) dynamics due to the CLT, whereas *μ* < 1 does not correspond to normalizable functions. In the present case of a large but finite *ℓ*_max_, if steps that end without a target detection outnumber considerably the truncations by targets encounters—as it happens under sparse conditions—then the heavy-tailed Lévy statistical properties of the move lengths distribution actually sustain for a huge number of steps, with ultraslow convergence to the CLT [112].

An appropriate statistical definition of the search efficiency [1, 49] is the ratio between the number of targets found and the total distance traversed during the search, *η* = *N*_found_/*L*_total_. Thus, *η* is equivalent to the inverse of the average distance traveled between successive encounters.

For the numerical simulations, each random search lasts until the searcher encounters *N*_found_ = 10^4^ targets. Averages are taken over 2.5 × 10^3^ runs, with each run corresponding to a landscape realization defined by a given set of parameters (*R*^(*p*)^, $l_{t}^{\left(p\right)}$, *β*, *γ*, etc.). In the next section we study some representative examples. In fact, we have also exhaustively tested many other different parameter sets, which actually led to the same qualitative conclusions discussed below.

### On the targets features and optimal strategies

In the present work we study non-destructive targets (allowing unrestricted revisits, see above). A trivial example would be a tree with plenty of fruits. An animal could get a fruit (the target) and leave the site (the tree location). Any time later, it can come back to that site and get a new fruit, which is effectively the same non-destructive target. Nonetheless, there are many other possibilities that could also be addressed, and we will mention a few of them in the following.

But first, we emphasize that a Lévy strategy means that a forager is following the specific rules described above. Thus, the distribution of step lengths is always given by Eq (5), with 1 < *μ* ≤ 3. Note then that the whole family of walks is characterized by a single parameter *μ*. An optimal Lévy search means that there exists a particular value of *μ* = *μ*_*opt*_, for which the searching efficiency is very high, actually the best value among all the possible *μ*’s. Since by varying *μ* we have a huge diversity of statistical features for the resulting walk patterns [1, 2], this simple Lévy strategy is ubiquitous in terms of distinct movement behavior (considering it is define by just an unique parameter).

The targets could be destructible, i.e., once a site is visited and the target is gathered, revisits to the now empty location are useless. Hence, for randomly distributed destructible targets, the optimal Lévy search strategy results from a ballistic-like dynamics, i.e., by setting *μ* → 1 (see, e.g., [1, 49, 150]). A more general situation is that of regenerative targets, in which a target site can become profitable again, but only after a certain regeneration (or delay) time *τ*_*d*_. Then, for distinct landscapes, the optimal value of *μ* (ranging from 1 to 2) would depend only on the specific value of *τ*_*d*_ [151, 152]. Lastly, we mention targets that are not all equal (e.g, representing different diet resources) and the forager is not looking only for quantity but also for diversity. In this case, it has been shown that the optimal *μ* can assume different values, depending on how the distinct types of targets are grouped together [153]. Incidentally, one possible solution [153] is *μ*_*opt*_ = 3/2 (or values around it), a Lévy foraging exponent experimentally observed in diverse contexts [24, 74, 154–158].

Therefore, the exact value of *μ*_*opt*_ might depend on the targets’ specificities. But the important fact is that the search optimization mechanisms and the step length distributions have the same structure. In other words, the basic trade-offs remain the same. In terms of evolution, this is a relevant fact because to respond to a transforming environment, an individual following a general Lévy walk behavior could simply change its *μ* value (e.g., to the optimal one corresponding to the targets new features). This ability represents a cheap adaptation. Thus, the discussions here for non-destructive targets would apply exactly in the same way for other target scenarios.

We observe that in some situations *μ*_*opt*_ is the same regardless of the targets’ features. For instance, for patchy landscapes the optimal foraging behavior for both destructive and non-destructive targets have been investigated in [81] and in the two cases one finds *μ*_*opt*_ ≈ 2. This result can be understood by noticing that the patchy regions are rich in resources, with a high inner density of targets. Thus, the consumption (or destruction) of a target found within a patch only slightly influences the searcher dynamics, as there is always another nearby target available for the forager.

In our simulations we consider only 2D environments (since these are computationally less time-consuming). Surely, many evidences of Lévy flight foraging behavior come from flying and swimming animals (see the many examples discussed and cited along the present work), i.e., in true 3D landscapes. However, we mention that the Lévy walk optimization process is essentially the same in 2D and 3D (e.g., refer to the numerical and analytical approach in [9, 159]). Therefore, our qualitative results here should also be fairly valid in 3D. Finally, we only mention a particular issue about 3D foraging strategies, for both short- and long-term search efficiencies. Optimal long-term searching strategies are independent of the foraging space. But this independence may not hold for the short-term component. There is the possibility that, in a 3D environment, the incorporation of long helical paths (i.e., as a short-scale searching strategy) [47, 160] improves the statistics of encounters by decreasing the probability of “missing” near targets, and/or by avoiding midterm curvilinear biases, or yet by reducing some energetic costs. This possibility remains an open question that would merit further consideration.

## Results

### Lévy as the optimal strategy for several distinct environments

**Fig 5:**
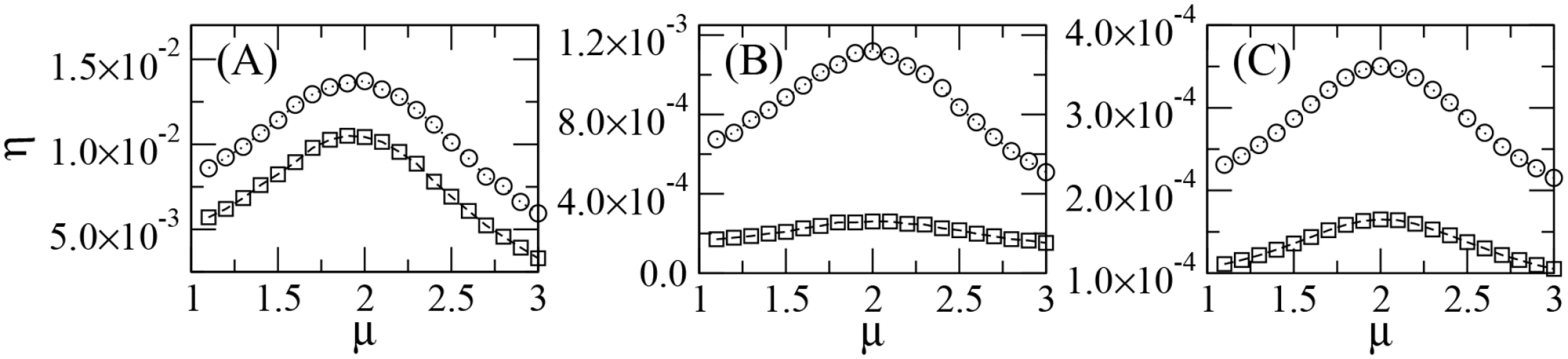
Search efficiency *η* vs. power-law exponent *μ* of Lévy searches for 10^4^ targets in fragmented landscapes. In the simulations, *N*_*p*_ = 10 (circles) and *N*_*p*_ = 5 (squares) heterogeneous patches contain a total of *N*_*t*_ = 10^4^ inner targets (see Methods section). In (A)-(C) the parameters determining the radii and average distances between inner targets are respectively set as in Fig 1(A)–1(C). Ballistic and Brownian limits correspond to *μ* → 1 and *μ* = 3, respectively. In all cases, the efficiency *η* is maximized for the superdiffusive dynamics with *μ*_opt_ ≈ 2.

Fig 5 displays the search efficiency *η* as a function of the power-law exponent *μ* in the cases of *N*_*p*_ = 5 and *N*_*p*_ = 10 heterogeneous patches with Euclidean distribution of targets (see Methods section). Results shown in Fig 5(A)–5(C) correspond [145], respectively, to configurations with patches of random sizes, random targets densities, and random sizes and target densities, which are exemplified in Fig 1(A)–1(C). Some search trajectories with different degrees of diffusivity are also shown in Fig 1(A)–1(C): nearly ballistic (*μ* = 1.1), superdiffusive (*μ* = 2.0), and Brownian (*μ* = 3.0).

We remark in Fig 5 that distinct degrees of heterogeneity achieved by varying the density of targets and number and size of patches do not affect the classical result *μ*_opt_ ≈ 2 for the optimal strategy, which has been also obtained in homogeneous environments [49, 161], as well as in landscapes with uniform patches [81]. Once the searcher leaves a patch, the detection of a new fragment is a task optimized by the ballistic strategy *μ* → 1 [145]. Nevertheless, this trend has to be balanced by the return to the patch just visited and an intensive local scanning of inner targets, which favor oversampling and are best promoted in diffusive strategies with *μ* → 3. Thus, the intermediate value *μ*_opt_ ≈ 2 emerges as a tradeoff between these opposite tendencies.

**Fig 6:**
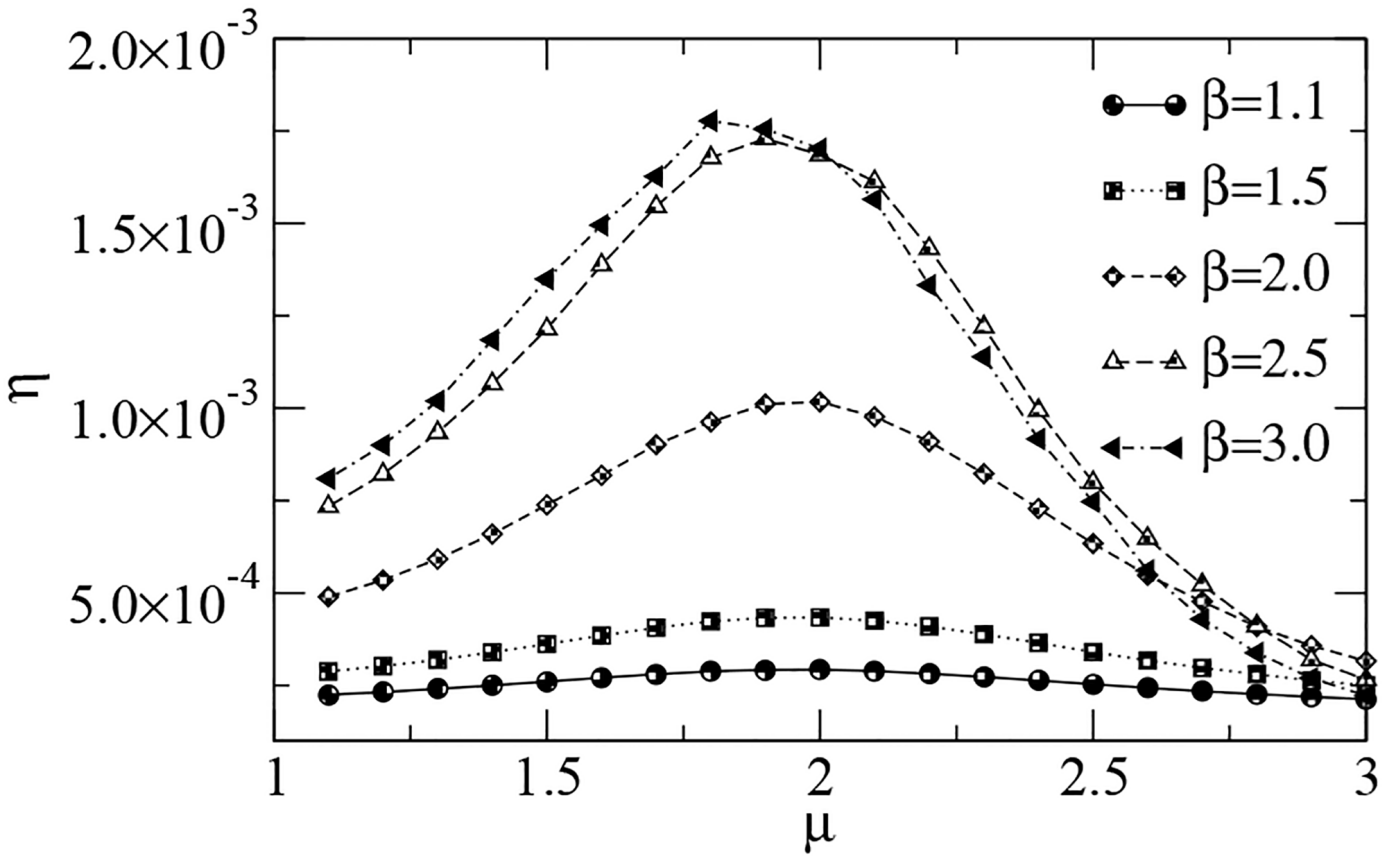
Search efficiency *η* vs. power-law exponent *μ* in Lévy dust distributions. The searcher detected 10^4^ targets in a landscape with Lévy dust distributions of *N*_*t*_ = 10^4^ targets (see Methods section). Parameters are set as in Fig 2. High clustering of targets and nearly homogeneous landscapes correspond to *β* = 3 and *β* = 1.1, respectively. In all cases, *η* is maximized for *μ*_opt_ ≈ 2, with a slight decrease in the optimal value (i.e. enhanced superdiffusion) observed as *β* → 3.

The efficiency of searches performed in Lévy dust distributions of targets is shown in Fig 6. Again, the value *μ*_opt_ ≈ 2 for the optimal strategy holds irrespective of the degree of clustering of targets driven by the exponent *β*. In fact, we notice that *μ*_opt_ is slightly lowered when clustering is high (e.g., *μ*_opt_ ≈ 1.8 for *β* = 3). Indeed, in landscapes similar to those depicted in Fig 4(C) and 4(D)—with a huge “open space” available —, once the searcher departs from the region plenty of targets it needs large steps (more likely for smaller *μ*) and enhanced superdiffusivity to come back. On the other hand, as seen in Fig 4(A), the homogeneous environment resembles the case with *β* = 1.1, for which *μ*_opt_ ≈ 2 is well known to apply [49]. The fact that a larger *β* implies a higher efficiency *η* means that landscapes containing a smaller occupied area with denser concentration of targets are generally more profitable than those with a larger occupied area of dispersed targets. Nevertheless, this might not be the case when other biological factors besides foraging are also taken into account along the searcher’s dynamics [81, 82, 145] (a nice example being that the risk of predation tends to favor smaller values of *μ*, see [82, 110, 162–164]).

**Fig 7:**
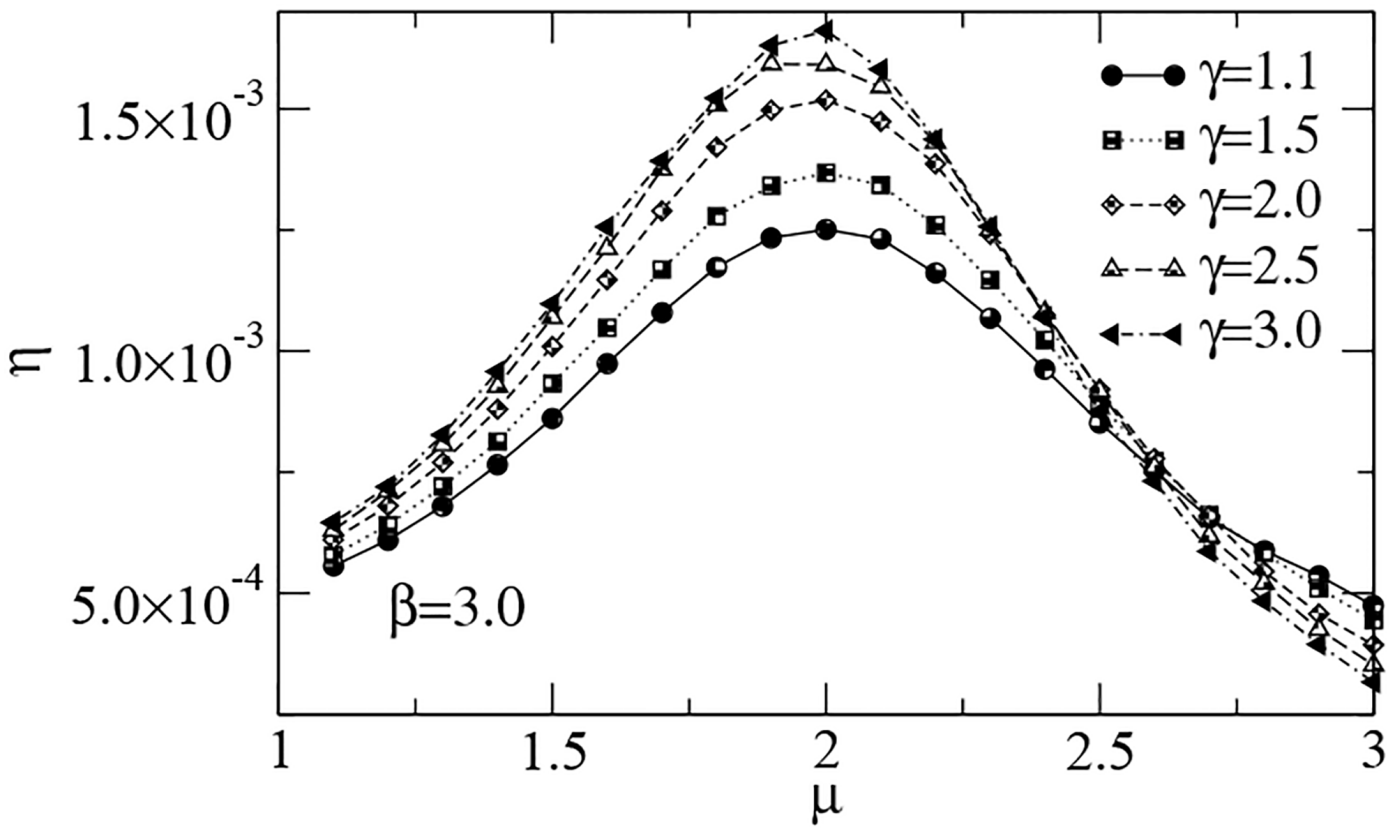
Search efficiency *η* vs. power-law exponent *μ* in fractal patches. The searcher detected 10^4^ targets in a landscape with Lévy dust distributions of *N*_*t*_ = 50000 targets in *N*_*p*_ = 50 patches (see Methods section). Parameters are set as in Fig 4. In all cases, *η* is maximized for *μ*_opt_ ≈ 2.

A similar scenario is found in Fig 7, in which the efficiency of searches in landscapes with Lévy dust distributions of both patches and inner targets is displayed. Here, the degree of clustering of the inner targets is fixed (*β* = 3), and results for several patch aggregation, driven by the exponent *γ*, are shown. The optimal search strategy, with *μ*_opt_ ≈ 2, is obtained once more. We also note that varying the value of *β* does not alter the qualitative behavior of the *η* vs. *μ* curves in this case.

Conceivably, the most common pattern of spatial distribution of resources in actual ecosystems is the patchy one (see, e.g., [82] and references therein). In this sense, by assuming a wide range of distributions of targets inside the patches, from highly fractal to fairly homogeneous (as *β* varies), and several degrees of patches aggregation (as *γ* varies), one is able to cover a broad diversity of search environments. Hence, it is a remarkable fact that in all these cases, and under the reasonable assumptions made in this work, essentially the same Lévy search strategy with a unique free parameter, *μ*, is able to optimize the search outcome. In the next section, we radically move away from the sparse-targets limit and investigate on a regime of searches in which the relative advantages of *μ* ≈ 2 search strategies are no longer so evident.

### The super-dense limit: Brownian searches as an emergent behavior

How significant is an optimal search strategy in a super-dense environment in which targets are so plentiful that long steps rarely occur? In this case, the key aspects determining efficient searches should be conceivably much different from those of the sparse regime. The reaction component of the search dynamics (e.g., the skills in getting the targets) becomes more relevant than the diffusion part of the process. As a consequence, the importance of extensive searching is reduced and one may anticipate that the relative advantage of strategies with *μ*_opt_ ≈ 2 tends to diminish. Thus, we now turn to the issue of how the preceding results obtained for generally sparse conditions are affected in *super-dense* homogeneous landscapes with very short average distance between targets, say *l*_*t*_ ≤ 5.

From the numerical perspective, in the super-dense limit the landscape construction and search dynamics are time consuming, moreover requiring extra technical attention. For instance, to avoid dynamical traps in the tight arrangement of targets we have constrained the distance between any two targets to be always greater than 1.1 (recall that *r*_*v*_ = 1). This condition prevents the searcher from artificially bouncing back and forth between two targets which are less than *r*_*v*_ away.

**Fig 8:**
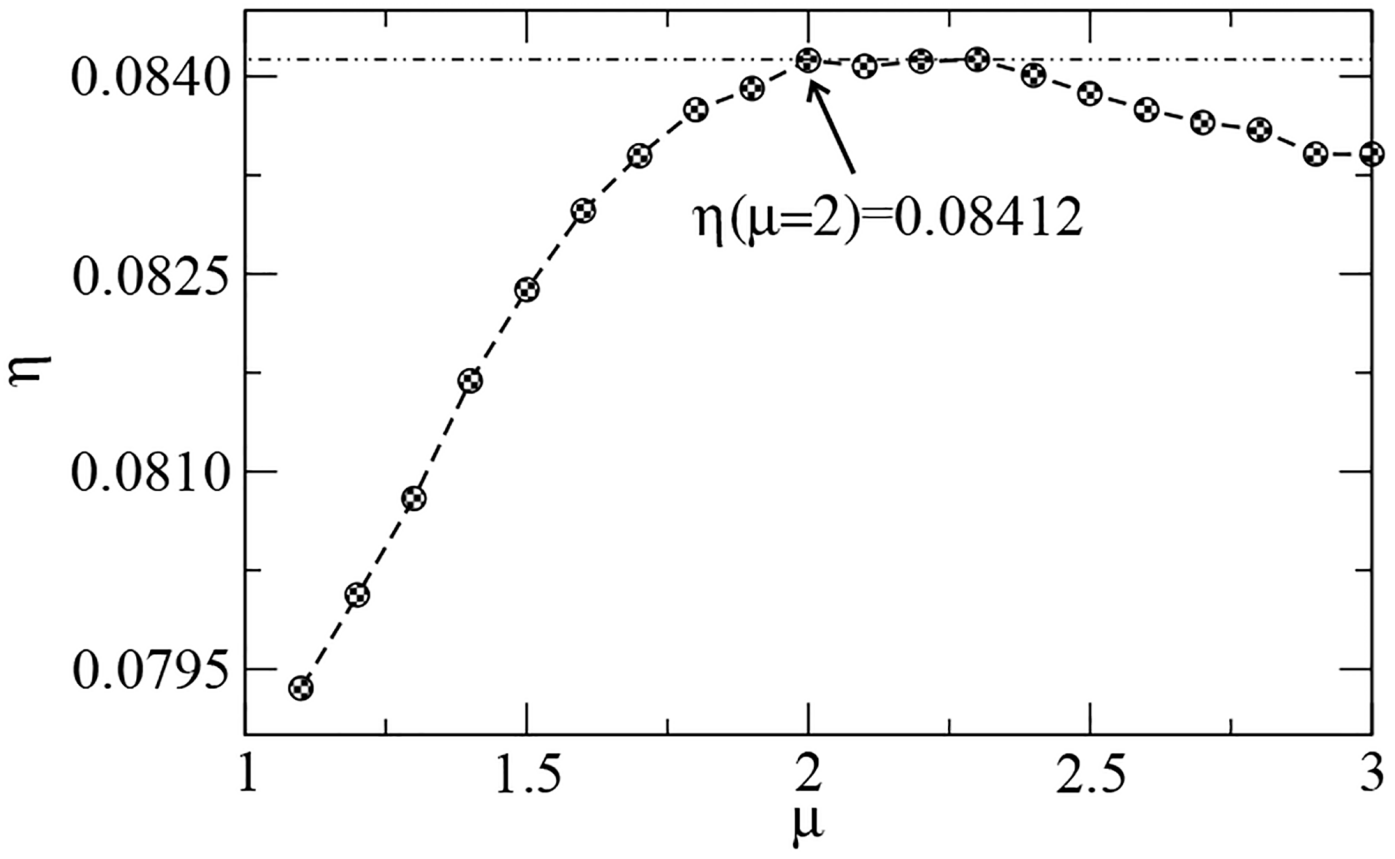
Search efficiency *η* vs. power-law exponent *μ* in a *super-dense* landscape. The simulations lasted for 10^4^ detected targets in a landscape with *l*_*t*_ = 5 and homogeneous distribution of *N*_*t*_ = 50000 targets. Note that *μ*_opt_ ≈ 2 is still the optimal value. However, in contrast with the sparse regime (see previous *η* vs. *μ* plots), less superdiffusive search walks (2 < *μ* < 3) and even diffusive ones (*μ* = 3) perform nearly as efficient as the optimal superdiffusive strategy.

The random search model is the same as considered in the previous sections. Fig 8 displays the efficiency *η* vs. the power-law exponent *μ* for *l*_*t*_ = 5, with averages taken over a smaller number (200) of landscapes due to the much longer simulations (in some cases, we have numerically checked that the qualitative results do not seem to alter if a larger number of averages is considered). The most evident finding is that less superdiffusive search walks (2 < *μ* < 3) and even diffusive ones (*μ* = 3) now perform nearly as efficient as the optimal strategy with *μ*_opt_ ≈ 2. Indeed, long steps *ℓ* ≫ *l*_*t*_, more likely to occur for small values of *μ*, are notably unnecessary in the super-dense scenario (see, e.g., [109]).

**Fig 9:**
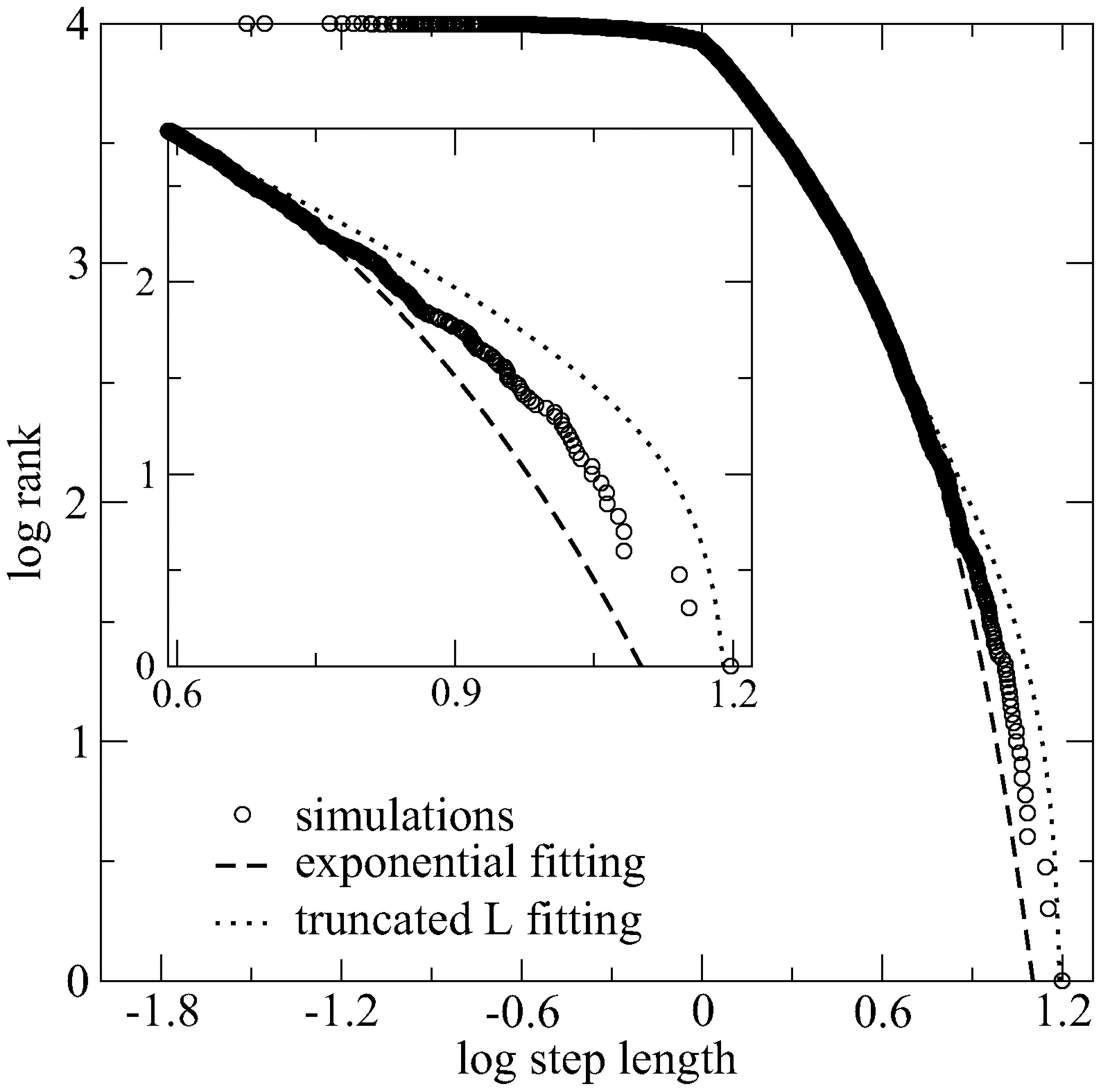
Output distribution of step lengths for a *μ* = 2.0 Lévy searcher in a *super-dense* landscape. In the simulations, *l*_*t*_ = 2.5 with *N*_*t*_ = 50000 targets homogeneously placed. The distribution takes into account the first 10^4^ search steps, including non-truncated moves that end up without detecting a target and also a relatively large number of truncated steps due to targets encounters. Numerical simulation data are represented by circles. Dashed and dotted lines are, respectively, best fits to Brownian-like exponential and truncated power-law pdfs. The inset details the large-steps regime. Statistical data inference (MLE and AIC methods) indicates that the output distribution of step lengths in the super-dense regime is not properly described by a superdiffusive power-law (Lévy-like) pdf. Instead, it shows the signature of a Brownian motion.

The effect of the high truncation rate by the encounter of targets in the super-dense landscape can be realized in Fig 9. We considered a *μ* = 2 Lévy search walk in a homogeneous environment with *l*_*t*_ = 2.5. In Fig 9 we show the output distribution of step lengths, which is the frequency of actual step sizes performed by the searcher during the full search. It computes non-truncated steps that end up without detecting a target, as well as the relatively large number of truncated moves due to targets encounters. Actually, the ratio of the number of truncated steps to the non-truncated ones decreases for larger *l*_*t*_ as $∼1/l_{t}^{(\mu -2)/2}$ in one-dimensional space [165], and decays even faster in two dimensions [166]. The output distribution of step lengths is thus expected to be strongly driven by the landscape properties in the case of super-dense regimes.

By focusing on the tail of the output distribution of step lengths (see inset of Fig 9), we can infer the quality of fits by a truncated power-law and a Brownian-like exponential function. Through the maximum likelihood estimation (MLE) method, we found that the log-likelihood of the truncated power-law (−2947) is larger (in absolute value) than that of the exponential distribution (−2938). Moreover, the Akaike information criterion (AIC) provides Akaike weights which equal 0 for the truncated power-law and 1 for the exponential. Therefore, the statistical analysis supports the conclusion that the output distribution of step lengths in a super-dense environment has the signature of a Brownian motion. Indeed, even the best-fit exponent for the truncated power-law output distribution (*μ* = 3.3) reflects the Brownian character of the optimal search walk.

**Fig 10:**
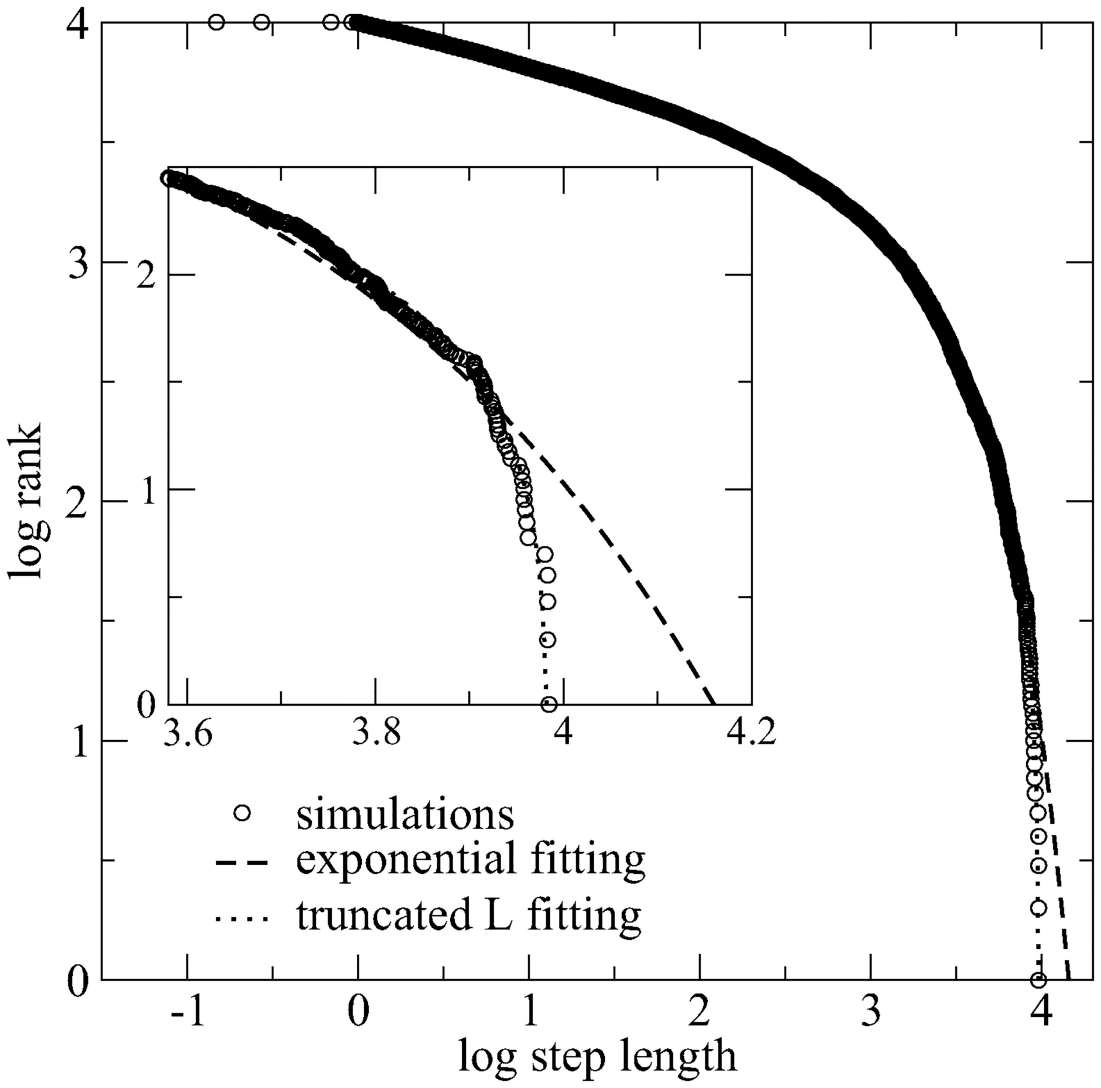
Output distribution of step lengths for a *μ* = 2.0 Lévy searcher in a *low-dense* landscape (*l*_*t*_ = 100). In the sparse regime, the number of truncated moves due to targets encounters is relatively low and long steps are much more frequent, if compared to the super-dense limit (see inset). Statistical data inference (MLE and AIC methods) indicates that the output distribution of step lengths in the low-dense regime is actually a power-law (Lévy-like), with best-fit exponent *μ* = 2.19 close to the original one.

For comparison, a similar study was performed under *low-dense* conditions (*l*_*t*_ = 100), Fig 10. In this case, the number of truncated steps is much lower, when compared to the super-dense limit. A *μ* = 2 Lévy search walk gives rise to an output distribution of step lengths which is still a truncated power-law, with best-fit value *μ* = 2.19 close to the original one. Indeed, the log-likelihood of the truncated power-law is −5571, with absolute value smaller than that of the exponential function (−5593). Also, the Akaike weight is 1 for the truncated power-law and 0 for the exponential, consistently with the findings in the low-dense regime.

## Discussion

The current debate on the *emergentist* vs. *evolutionary* origin of animal foraging is one of the most important issues in behavioral ecology [50, 51]. In its heart lies the paradigmatic Lévy flight foraging hypothesis, proposed more than a decade ago [1, 49]. Lévy strategies are robust [145, 152] in optimizing random search processes—or at least to yield advantageous outcomes—under conditions of scarce information availability and sparse targets, a common situation in actual ecosystems. In this sense, the *evolutionary* scenario states that organisms would have evolved via natural selection to exploit these advantageous distributions of move lengths.

On the other hand, in the *emergentist* view a Lévy foraging pattern would arise from complex interactions between the searcher and the environment. In other words, it would depend on the exact mechanisms through which the landscape features influence the individuals’ actions.

As in any scientific query, the full picture must rely on accurate empirical data together with suitable analysis based on solid conceptual interpretation [167]. Hence, theoretical arguments and proper models are essential in helping to identify the fundamental features of the process, leading to definite conclusions. Thus, statistical physics, and more specifically random search theory, can contribute in an important way to address the problem, exactly the approach followed in the present work.

We have studied through a reaction-diffusion algorithm the impact of landscape diversity on efficient search dynamics. Remarkably, *the same* optimal solution in the broad class of Lévy walks arose in a large collection of assorted environments, differing in diverse aspects such as the density of targets, the number and size of patches, the degree of heterogeneity, fragmentation and fractal dimension of the Lévy dusts, provided the targets density is sparse and the searcher’s information is restricted to its vicinity.

We have also reported that deviations from the Lévy search dynamics can be effectively observed in plentiful landscapes (e.g., from the actually measured step lengths distribution, Fig 9), thus indicating that Lévy search walks is certainly not ubiquitous in Nature (although common). In this case, the emergent optimal strategy becomes strongly driven by the encounter interactions with the complex environment. A number of non-optimal strategies perform almost as efficient as the optimal one, and the pressure towards greater resource rewards obtained through the correct choice of optimal or nearly optimal strategies is then reduced [109].

Interestingly, in this high-dense regime a Brownian distribution of step lengths emerges due to the frequent truncation of steps, even if a Lévy pdf is considered as the input distribution, i.e., the inherent dynamics. Indeed, the plentiful availability of resources naturally induces an interaction with the landscape through the finding and consumption of target sites (it is relevant to mention that these analyzes corroborate similar results and conclusions presented in [21]). Conversely, changes in the environment’s features towards a depletion regime can drastically decrease the targets encounters, and thus an already existing (“built-in”) search Lévy behavior—conceivably developed through natural evolution—would help to improve the chances of successful foraging without the necessity of drastic switching mechanisms.

At this point some words of caution are in order. Since the 1990s, the empirical demonstration of the occurrence of Lévy-type probability distributions in stochastic phenomena has notably grown, with a great diversity of examples raising both from biological as well as non-biological systems [1, 2]. In this sense, it seems clear that other mechanisms rather than the Lévy flight foraging hypothesis can be also responsible to generate such heavy-tailed distributions. Actually, depending on the specificities of the problem, even in biological systems Lévy distributions may arise in contexts outside the domain of efficient random searches. For instance, we mention that the flight distances by shearwaters follow a power-law pdf with exponent *μ* ≈ 3/2, which can be attributed to the olfactory maps the birds use for navigation [26], not related to search strategies. Also, the emergent-induced Lévy paths by bumblebees may arise as a consequence of the rejection of a great number of flowers previously marked by other individuals with repellent scent [65]. Other examples include the movement of seeds, jellyfish, and swarming bacteria, and even protein motors and DNA [60]. Therefore, in this sense it is thus clear that our results favoring an evolutionary view implied by the Lévy flight foraging hypothesis address the specific context of the evolved optimal probabilistic foraging strategies.

Certainly, a great deal of theoretical and experimental effort is still necessary before some key questions in foraging can be answered. In the context of the *emergentist* vs. *evolutionary* debate, our findings do not definitely overturn the emergentist hypothesis. Nonetheless, the robustness of Lévy walks—showing search efficacy in a wide variety of landscapes—favors the hypothesis of an evolutionary origin for different motor programs that could produce Lévy walk or similar statistical properties (see the section: Important features of efficient foraging strategies).

Note that in principle the emergent view supports specific (rather than general) optimal responses according to the landscape characteristics. But a caveat is that such dedicated strategies would not be plastic, hence not evolutionarily sustainable at long time scales. An alternative *emergentist* scenario would be the one in which proper (e.g., superdiffusive) movement features would arise modulated by searcher-environment interactions in each particular ecological network [168]. Despite existing a diverse range of putative generative mechanisms for Lévy walk signatures to emerge (as we have discussed above), a key point is that different environments may induce very different interaction mechanisms. Moreover, many of them might give rise to properties out of the Lévy (and alike) optimized regimes. Thus, without an universal evolutionary pressure it becomes more difficult to accept landscape-dedicated species-specific advection diffusion responses systematically yielding akin Lévy-like behaviors. For instance, it has been proposed that landscapes act as modulators [169], strongly affecting local patterns of biodiversity. The relations between a group of species in habitat A can be considerably distinct from those for the same species but in habitat B [170]. Also, trophic interactions (e.g., the predator-multiprey competition) can be modified by the presence of heterogeneities in the environment [171, 172]. Hence, the implausibility of retaining Lévy strategies in complete distinct environments without any underlying evolutionary pressure has motivated the idea of a general Hierarchical Lévy Flight Foraging Hypothesis [50], of which examples in [60] and [79] are potential concrete cases.

In conclusion, under the specific ecological assumptions assumed throughout our discussions, the *emergentist* hypothesis (in its more restricted form) seems to be a limited framework in face of the present theoretical analysis and when confronted with the available empirical data in the literature. Importantly, many of the points raised here eventually may be answered only through combining: accurate experimental data in controlled conditions, animals with restrained (and known) internal motivations, and well designed modeling [52, 167]. We hope that this work can stimulate progress and promote advances in the exciting field of foraging ecology.
